# Systemic Resolvin E1 (RvE1) Treatment Does Not Ameliorate the Severity of Collagen-Induced Arthritis (CIA) in Mice: A Randomized, Prospective, and Controlled Proof of Concept Study

**DOI:** 10.1155/2019/5689465

**Published:** 2019-10-31

**Authors:** Rafael Scaf de Molon, Rogier M. Thurlings, Birgitte Walgreen, Monique M. Helsen, Peter M. van der Kraan, Joni Augusto Cirelli, Marije I. Koenders

**Affiliations:** ^1^Department of Diagnosis and Surgery, School of Dentistry at Araraquara, Sao Paulo State University-UNESP, Araraquara, SP, Brazil; ^2^Department of Rheumatology, Radboud University Medical Center, 6500 HB Nijmegen, Netherlands

## Abstract

Specialized proresolving mediators (SPRM), which arise from n-3 long-chain polyunsaturated fatty acids (n-3FA), promote resolution of inflammation and may help to prevent progression of an acute inflammatory response into chronic inflammation in patients with arthritis. Thus, this study is aimed at determining whether systemic RvE1 treatment reduces arthritis onset and severity in murine collagen-induced arthritis (CIA) and spontaneous cytokine production by human rheumatoid arthritis (RA) synovial explants. 10-week-old DBA1/J male mice were subjected to CIA and treated systemically with 0.1 *μ*g RvE1, 1 *μ*g RvE1, 5 mg/kg anti-TNF (positive control group), PBS (negative control group), or with a combination of 1 *μ*g of RvE1 plus 5 mg/kg anti-TNF using prophylactic or therapeutic strategies. After CIA immunization, mice were treated twice a week by RvE1 or anti-TNF for 10 days. Arthritis development was assessed by visual scoring of paw swelling and histology of ankle joints. Moreover, human RA synovial explants were incubated with 1 nM, 10 nM, or 100 nM of RvE1, and cytokine levels (IL-1*β*, IL-6, IL-8, IL-10, INF-*γ*, and TNF-*α*) were measured using Luminex bead array. CIA triggered significant inflammation in the synovial cavity, proteoglycan loss, and cartilage and bone destruction in the ankle joints of mice. Prophylactic and therapeutic RvE1 regimens did not ameliorate CIA incidence and severity. Anti-TNF treatment significantly abrogated signs of joint inflammation, bone erosion, and proteoglycan depletion, but additional RvE1 treatment did not further reduce the anti-TNF-mediated suppression of the disease. Treatment with different concentrations of RvE1 did not decrease the expression of proinflammatory cytokines in human RA synovial explants in the studied conditions. Collectively, our findings demonstrated that RvE1 treatment was not an effective approach to treat CIA in DBA1/J mice in both prophylactic and therapeutic strategies. Furthermore, no effects were noticed when human synovial explants were incubated with different concentrations of RvE1.

## 1. Introduction

Rheumatoid arthritis (RA), an immunologically driven chronic disorder, is characterized by infiltration of synovium by activated inflammatory cells, synovial hyperplasia, and progressive destruction of the cartilage and bone. These features result in long-term joint damage, chronic pain, loss of function, and progressive disability [1, 2] RA affects up to 1% of the population worldwide, is three times more prevalent in women, and is associated with significant comorbidities (cardiovascular illness, skeletal disorders) [3], socioeconomic burden, and mortality [4]. The exact etiology of RA is still poorly understood albeit it is hypothesized that the development of RA is dependent on the complex associations between environmental factors (smoking, microbiome), genetic background (HLA-DRB1 gene), and hormonal and infectious risk factors [5–7], resulting in the formation of autoantibodies and RA onset.

An exacerbated immune response from the activation of immune and resident synovial cells is responsible for most of the cartilage damage observed in RA, whereas osteoclastic activation plays a major role in bone destruction [8, 9]. Several treatments for RA have been developed and comprise corticosteroids, nonsteroidal anti-inflammatory drugs (NSAIDs), disease-modifying antirheumatic drugs (DMARDs), biologic agents (like tumor necrosis factor or interleukin inhibitors), and JAK inhibitors [10]. All treatment strategies are aimed at reducing periarticular inflammation, limiting joint destruction, and improving health-related quality of life.

Recent key findings in the mechanisms of inflammation hold promise for the development of a new treatment for RA using proresolving mediators. Resolution of inflammation, which was considered a passive process, has been found to involve active biochemical programs that enable inflamed tissue to return to homeostasis [11, 12]. Omega-3 fatty acid-derived molecules, termed proresolving mediators, are involved in this process. The endogenous proresolving mediators are not immunosuppressive but function in the resolution of inflammation by activating specific mechanisms to promote homeostasis [11, 12]. Shortly, they selectively stop neutrophil infiltration, stimulate recruitment of monocytes (without elaborating proinflammatory mediators), activate macrophage phagocytosis of microorganisms and apoptotic cells, and stimulate expression of molecules involved in antimicrobial defense. As a result, a shift in inflammatory response to a shorter resolution interval occurs, which may help to prevent progression of an acute inflammatory response into chronic inflammation [13].

Proresolvin mediators, a novel family of lipid mediators including RvE1 and RvD1, show remarkable potency in treating disease conditions associated with inflammation, including inflammatory pain [14, 15], periodontal disease (PD) [16–19], bone preservation [20], and osteoarthritic pain [21]. This is because proresolution molecules promote uptake and clearance of apoptotic cells as well as microbes by macrophages in inflamed tissue and stimulate antimicrobial activities of cells [22]. Moreover, RvE1 modulates osteoclast differentiation and bone remodeling by direct actions on the bone, rescuing OPG production and restoring a favorable receptor activator of NF-*κ*B ligand/OPG ratio [20].

Oral application of the proresolving mediator resolvin E1 (RvE1) was shown to prevent onset and progression and even induce periodontal regeneration in a rabbit model of PD [16, 17]. RvE1 binds to the chemerin 23 and BLT1 receptors that are expressed by a range of stromal, innate, and adaptive immune cells. RvE1 reduced PD by inhibition of proinflammatory mediators and decreased bone loss. In addition, treatment with RvE1 resulted in reduction in the systemic inflammatory markers C-reactive protein and IL-1beta [16]. Of importance and in contrast to immunosuppressive drugs, RvE1 increased the clearance of PD-associated bacteria [17, 18]. This finding suggests that PD-associated bacteria actively direct the protective bactericidal immune response into a dysfunctional state, which may be reversed by proresolving therapy, but not by immunosuppressant. Of clinical relevance, an RvE1 mimetic is currently in clinical testing for a topical treatment of dry eye [23].

Despite the differences in the etiologies of RA (autoimmune-driven disease) and PD (infection-driven disease), the role of citrullination and autoantibody response and the pivotal role of oral bacteria and inflammation mechanistically link these two conditions [24, 25]. In this context, it is clinically highly relevant to investigate if RA disease activity can be diminished by systemic treatment using RvE1. In contrast to proresolving mediators like RvE1, current RA treatments, besides resulting in inadequate responses in up to 30% of the RA patients, have serious immunosuppressive side effects and are associated with adverse effects on the protective antibacterial immune response. Thus, the aim of this study was to investigate the effects of systemic administration of RvE1 in murine collagen-induced arthritis (CIA).

## 2. Materials and Methods

### 2.1. Animal Care

10-week-old DBA1/J male mice were obtained from Janvier-Elevage (Le Genest Saint Isle, France). Mice were housed in filter-top cages under standard conditions with controlled temperature (22-25°C) and humidity and with a 12 h light/dark cycle in separated and appropriated cages. They received free access to commercial chow and water ad libitum. The experimental protocol (#2015-0066-002) was approved by the local Institutional Animal Ethics Committee at the Radboud University Medical Center, Nijmegen, The Netherlands. The protocol followed all recommendations of the ARRIVE (Animal Research: Reporting in Vivo Experiments) guidelines for the execution and submission of studies in animals [26].

### 2.2. Preparation of RvE1 and Anti-TNF

Solution of RvE1 (Cayman Chemicals) was prepared in ethanol and kept in a -80°C freezer until its use. On the day of experiments, aliquots of RvE1 (0.1 *μ*g and 1 *μ*g) were dissolved according to the literature [15] and immediately injected via intraperitoneal (i.p.) injection in the mice. The TNF-*α* inhibitor etanercept (Enbrel®—5 mg/kg; Pfizer, New York, NY, USA) was injected i.p. following a previously published article [27]. Animals received RvE1 or anti-TNF treatment twice a week after the booster injection for 10 days.

### 2.3. Induction of CIA

Bovine type II collagen (CII) was prepared as described previously [28]. CII was diluted in 0.05 M acetic acid to a concentration of 2 mg/ml and emulsified in equal volumes of Freund's complete adjuvant (2 mg/ml of Mycobacterium tuberculosis strain H37Ra; both from Difco). The DBA/1J mice were immunized intradermally at the base of the tail with 100 *μ*g of CII. On day 21, mice received an i.p. booster injection of 100 *μ*g of CII dissolved in PBS, and the onset of arthritis usually occurred a few days after the booster injection. Mice were carefully examined 3 times per week for the visual appearance of arthritis in the peripheral joints, and scores for disease activity were given as previously described [29].

### 2.4. RvE1 and Anti-TNF Treatment

The effects of RvE1 and anti-TNF in CIA mice were evaluated using two treatment strategies: (1) prophylactic (before disease onset), in which i.p. injections of 0.1 *μ*g RvE1 (*n* = 10), 1 *μ*g RvE1 (*n* = 10), 5 mg/kg anti-TNF (*n* = 10), or a combination of 1 *μ*g RvE1 plus 5 mg/kg anti-TNF (*n* = 10) were performed in immunized, nonarthritic mice twice a week from the day of booster injection until 10 days later, and (2) therapeutic (after the onset of disease), in which i.p. injections of 1 *μ*g RvE1 (n = 10) were performed in arthritic mice starting 8 days after CIA booster and twice a week until the end of experiment (after 7 days of treatment). The control group (*n* = 10 for each prophylactic and therapeutic strategies) was injected (i.p.) twice a week with phosphate-buffered saline (PBS) solution. The dose of RvE1 and anti-TNF treatment were based on previously published protocols [19, 27].

### 2.5. Clinical Assessment of CIA

Mice were macroscopically scored for arthritis severity 3 times a week from day 14 after the first immunization until the time of sacrifice using a previously standardized arbitrary scoring system [30, 31]. The scores were based on a 0-2 scale per paw according to changes in redness and swelling in the digits or in other parts of the paws, in which 0 represents no joint swelling and 2 severe swelling of entire paw. The scores for both front and hind paws were totaled for each mouse (with a maximum possible score of 8 for each mouse).

### 2.6. Animal Euthanization and Analyses

All animals were euthanized by cervical dislocation at the end of the experimental period (10 days after the booster injection). Animals from the therapeutic group were sacrificed three days earlier due to the severity of arthritis (after 7 days of treatment). The ankle joints were removed and immediately fixed in 4% paraformaldehyde.

### 2.7. Histologic Analysis

Ankle joints were isolated and fixed in 10% formalin for 4 days, thereafter decalcified in 5% formic acid, and subsequently dehydrated and paraffin embedded; 7 mm thick semiserial sections, spaced 140 mm apart, were obtained, mounted on Super-Frost slides (Menzel-Glaser), and stained with hematoxylin and eosin (H&E) for inflammation and cartilage and bone destruction evaluation and with Safranin-O for proteoglycan depletion evaluation.

Briefly, infiltration of cells was scored in a scale of 0 to 3, depending on the amount of inflammatory cells in the synovial cavity and synovial lining (0 = no cells, 1 = mild cellularity, 2 = moderate cellularity, and 3 = maximal cellularity) with steps of 0.25. Chondrocyte death was distinctly graded on a scale of 0 to 3, ranging from the nonappearance of dead chondrocytes (empty lacunae) to a complete loss of chondrocytes in the cartilage. Cartilage and bone erosions were both scored on a scale of 0 to 3 ranging from no damage to complete structural loss. Proteoglycan depletion was scored on a scale of 0 to 3 ranging from complete red staining of the superficial cartilage to a complete loss of red staining of the superficial cartilage zone. All the evaluation parameters were based on previously published articles [30, 32].

### 2.8. Human RA Synovial Explant Assay

Synovial tissue specimens of six RA patients were obtained during joint replacement surgery (knee, elbow, and shoulder). To confirm synovial origin, characterized by the presence of an activated synovial lining, representative tissue samples were embedded in OCT and 5 *μ*m cryosections were cut and subsequently stained by H&E. Punch biopsies of Ø 3 mm were randomly allocated to the culture wells and cultured in the presence or absence of RvE1 (1, 10, 100 nM) in a 96-well plate in 200 *μ*l RPMI 1640 medium, supplemented with 10% fetal calf serum, sodium pyruvate, 100 units/ml penicillin, and 100 mg/ml streptomycin, for 24 hours. Then, supernatants were harvested and centrifuged for 5 minutes at 3000 RPM at room temperature. Supernatants were stored at -20°C until cytokine detection by Luminex. Assays were performed in quadruplicate. All patients provided informed consent, and the Medical Ethics Committee of Radboud University Medical Center Nijmegen approved the study protocol.

### 2.9. Luminex

Supernatants from the explant culture were collected for cytokine detection. Biopsies were cultured without or with RvE1. Then, cytokine levels were determined using Luminex bead array technology. Cytokines were measured in 50 *μ*l of culture medium for 6 cytokines of interest: IL-1*β*, IL-6, IL-8, IL-10, INF-*γ* and TNF-*α*. Cytokine levels were measured by Luminex multianalyte technology, using the Bio-Rad Bio-Plex™ 200 System with magnetic beads, in accordance with the manufacturer's protocols (Bio-Rad).

### 2.10. Mass Spectrometry

A Thermo Finnigan LCQ Fleet ESI ion-trap mass spectrometer, which is equipped with a Shimadzu HPLC (details follow with respect to column and program) and a PDA detector, was used to separate organic compounds and record low-resolution mass spectra. The analysis of a blanco (EtOH) and RvE1 was performed.

### 2.11. Statistical Analysis

Analyses were performed using GraphPad Prism Software (GraphPad 7 Software, Inc., La Jolla, CA). Group measures were expressed as mean and the standard error of the mean (SEM). Data normality was assessed by D'Agostino and Pearson normality test. Statistical significance was assessed using one-way analysis of variance (ANOVA) followed by Tukey's post hoc test for multiple comparisons among groups in the prophylactic strategies. Data between groups in the therapeutic strategies were compared using Student's *t*-test. Results with *P* < 0.05 were considered statistically significant.

## 3. Results

### 3.1. Chemical Stability of RvE1

**Figure 1:**
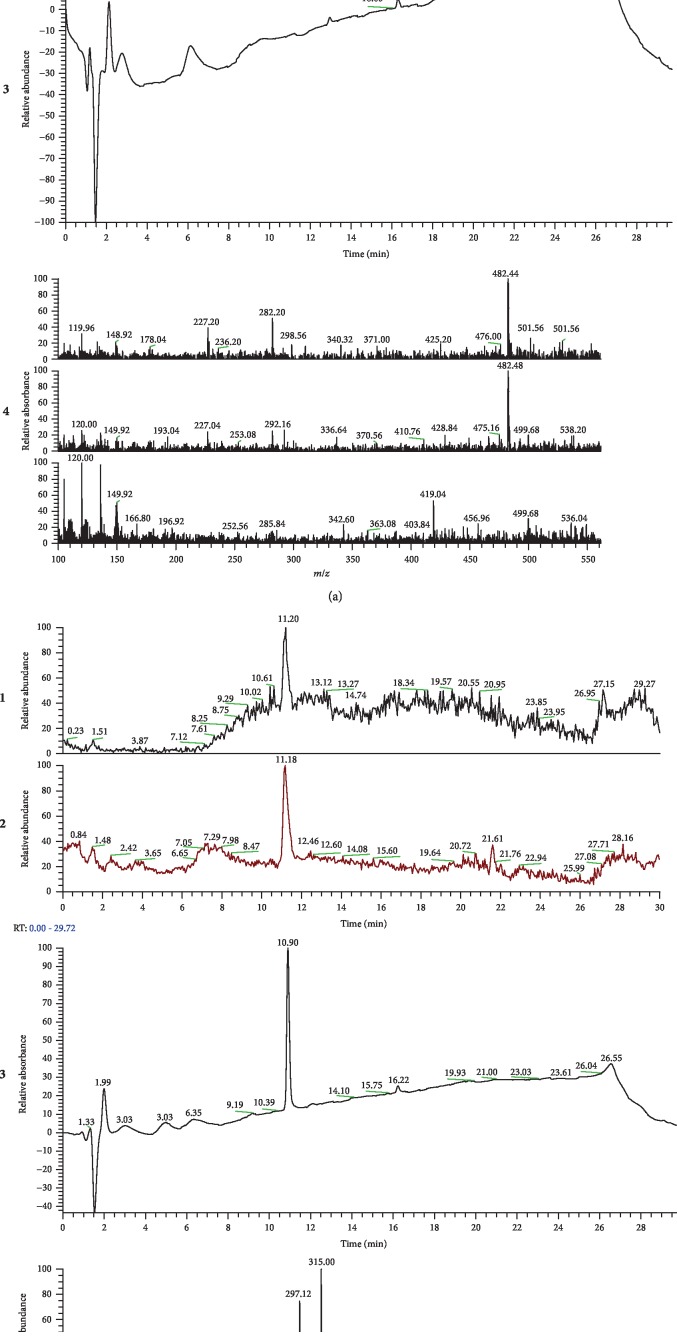
Mass spectrometry of (a) blanco (control) and (b) RvE1 to evaluate the UV spectrum of RvE1 and its integrity. On the left panel (a1–4) are the control samples (blanco—EtOH) and the right panel (b1–4) are the RvE1 samples. (a, b) 1 and 2 are negatively and positively charged analyses, respectively. (b3) shows the UV spectrum of RvE1: the first wave is the time of injection (1.33-1.99), and the peak of RvE1 is around 10.90 minutes later. (b4) shows further analyses of the peaks 11.20 and 11.18 evidencing more peaks of RvE1. These analyses demonstrated the chemical stability of the RvE1.

Resolvin E1 is light, pH and oxygen sensitive that can lead to decomposition of inactive products. To validate the chemical stability of RvE1, we have performed mass spectrometry to confirm that there was no degradation of RvE1 before injection in the animals. Our findings revealed that RvE1 compound was intact before injection (Figures 1(a) and 1(b)). The UV spectrum of RvE1 showed that after 10.9 minutes after the injection in the animals (1.33-1.99), the RvE1 spectrum reached its peak (Figure 1(b)). Further analysis of the peak (11.20) showed more peaks in 3 different forms: M-H-2H2O, M-H-H2O, and M+Na. The analysis of the peak (11.18) demonstrated a nice clean peak of RvE1 minus a hydrogen, M-H. These findings indicate that the RvE1 was stable before injection (Figure 1(b)).

### 3.2. RvE1 Treatment Does Not Affect Collagen-Induced Arthritis Incidence and Severity

**Figure 2:**
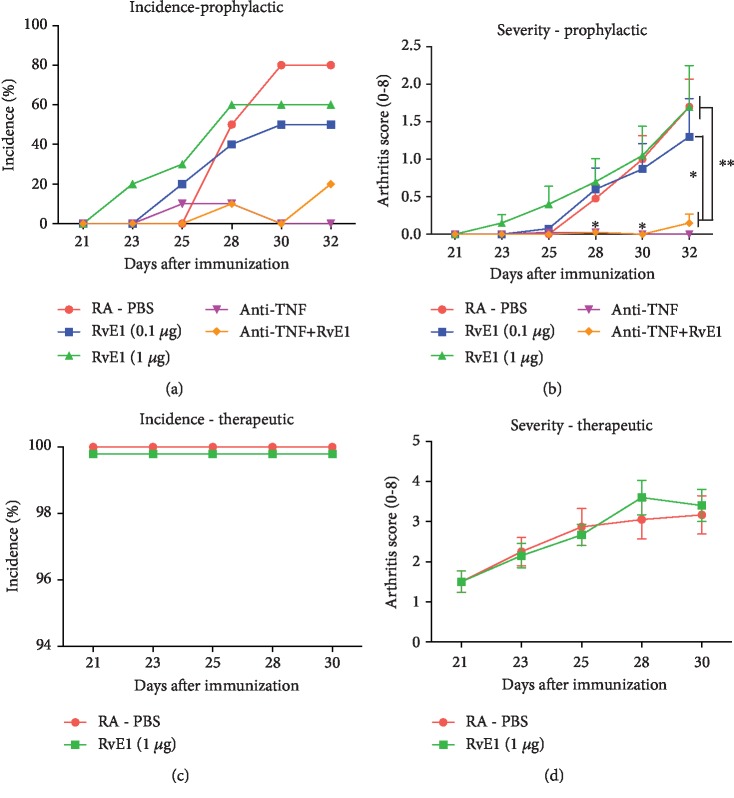
(a, b) Prophylactic treatment with RvE1 starting with negative mice at day 21 and (c, d) therapeutic treatment with RvE1 starting after CIA development (all positive mice). Arthritis incidence and severity scores (0–2 per paw) of DBA1/J male mice treated with low (0.1 *μ*g) and high dose (1ug) of RvE1, PBS, and 5 mg/kg anti-TNF and with a combination of 5 mg/kg anti-TNF plus 1 *μ*g RvE1. Data were expressed as the mean ± standard error of the mean (SEM). One-way ANOVA followed by Tukey's posttest was used to assess the differences among the 4 groups. Student's *t*-test was used to access the differences between the 2 groups. Differences were considered significant at *P* < 0.05. ^∗^Statistically significant difference from indicated groups (*P* < 0.001). ^∗∗^Statistically significant difference from indicated groups (*P* < 0.0001).

To investigate whether RvE1 treatment reduces CIA incidence and severity, mice were macroscopically scored for both prophylactic and therapeutic regimens. In the prophylactic regimen, immunized mice in the PBS and RvE1 groups demonstrated higher incidence of CIA development compared to mice treated with the TNF-*α* inhibitor. Treatment with anti-TNF almost completely abrogated the development of CIA (Figure 1(a)). No additional effect of the combination of anti-TNF plus RvE1 could be observed compared to TNF blocking alone. As expected, the severity of CIA in animals treated with anti-TNF was statistically significantly lower compared to the other groups treated with PBS and RvE1 (Figure 2(b)). No differences were found comparing the PBS control group and both dosages of RvE1 for the incidence and severity of arthritis (Figures 2(a) and 2(b)).

In the therapeutic regimen, without the TNF-*α* inhibitor and the low dose of RvE1 groups, all mice showed a progressive CIA development (Figures 2(c) and 2(d)). Comparing the PBS and RvE1 groups, no significant differences were found regarding CIA severity; i.e., RvE1 treatment was not able to slow down CIA progression (Figure 2(d)).

### 3.3. Histological Arthritis Scores Were Not Improved by RvE1 Treatment

**Figure 3:**
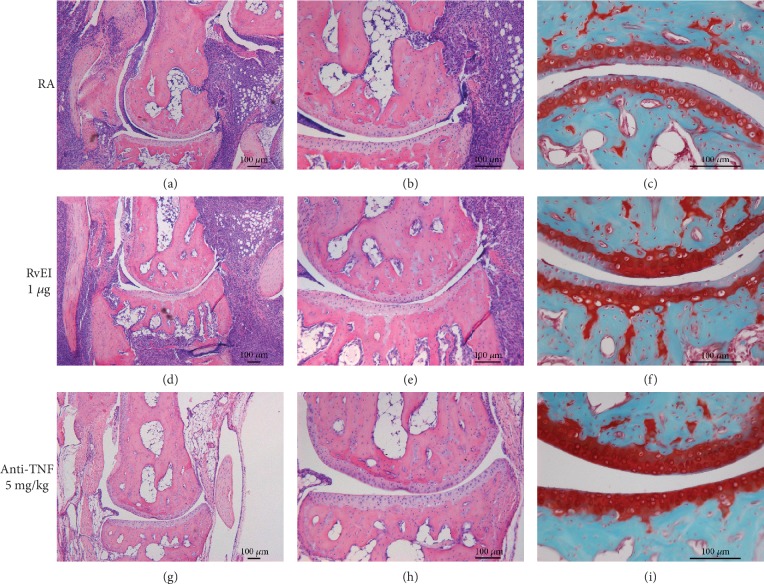
Histological characteristics from the prophylactic regimen. Representative H&E and Safranin-O-stained slides from the PBS (a–c), 1 *μ*g of RvE1 (d-f), and anti-TNF groups (g-i). The sections clearly show (a, b, d, e) the influx of inflammatory cells in the ankle joints of CIA mice. Moreover, bone resorption and cartilage destruction were also observed. In the Safranin-O-stained slides (c, f), proteoglycan depletion was noted. Original magnification of 10x, 20x, and 50x. Images from the anti-TNF-treated mice stained with H&E (g, h) and Safranin-O (I) illustrating decreased synovial inflammation, cartilage destruction, and bone erosion and normal proteoglycan layer.

**Figure 4:**
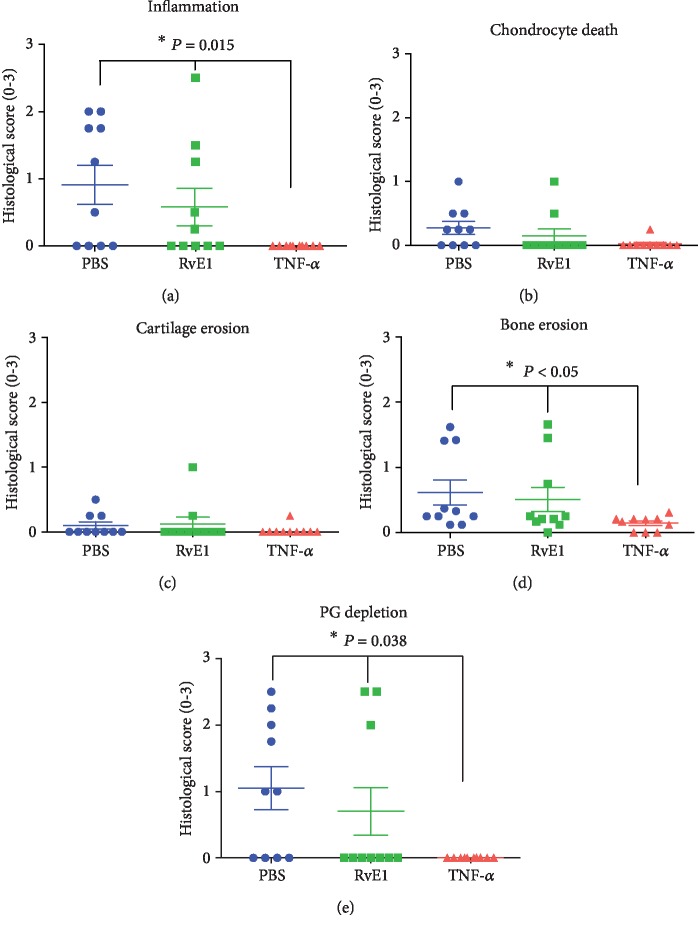
Quantification of histological findings—prophylactic. Histopathologic scores of synovial inflammation (a), chondrocyte death (b), cartilage erosion (c), bone erosion (d), and proteoglycan depletion (e) after PBS, RvE1, and anti-TNF treatment after induction of collagen-induced arthritis. One-way ANOVA followed by Tukey's posttest was used to assess the differences among the groups. Differences were considered significant at *P* < 0.05. ^∗^Statistically significant difference from the PBS and RvE1 groups.

Descriptive and quantitative histological analysis in the prophylactic strategy revealed severe influx of inflammatory cells in the ankle joints of mice treated with PBS and RvE1 (Figures 3(a)–3(e)). In these two groups, bone erosion, cartilage destruction, and PG depletion were also observed in contrast to the group treated with TNF blocking. (Figures 3(c) and 3(f)). In line with the macroscopic scores, mild inflammation and no damage to the cartilage or the bone were noted in animals treated with anti-TNF (Figures 3(g)–3(i)). To further confirm the descriptive analysis, quantification of histological findings was measured. In the prophylactic regimen (Figures 4(a)–4(e)), induction of CIA in immunized mice treated with PBS and high dosage of RvE1 triggered significant cell influx to the synovial cavity and increased PG depletion. The anti-TNF group resulted in a significant reduction of inflammation (*P* = 0.015), bone erosion (*P* < 0.05), and PG depletion (*P* = 0.038) compared to PBS- and RvE1-treated mice. (Figures 4(a)–4(e)). No differences were found comparing PBS-treated mice with a high dose of RvE1 treatment (1 *μ*g) for all the parameters measured. No differences were found among the groups for chondrocyte death and cartilage erosion (Figures 4(b) and 4(c)).

**Figure 5:**
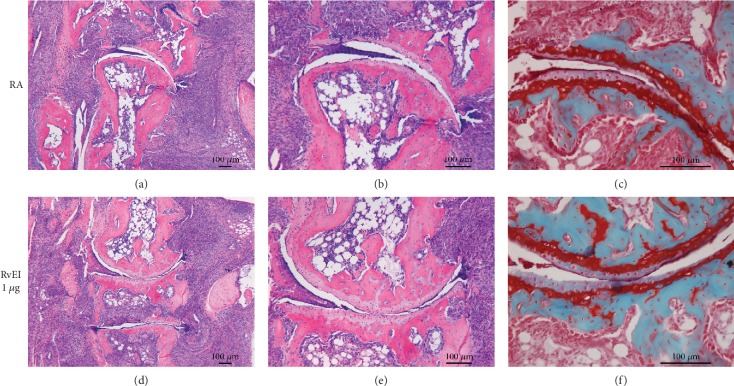
Histological characteristics from the therapeutic regimen. Representative H&E and Safranin-O-stained slides from the PBS group (a–c) and from the high-dose RvE1 group after RA development (d, f). In this strategy, all mice showed clinical signs of arthritis at the start of treatment. As expected, CIA was very pronounced in the absence of treatment (a–c). In the Safranin-O-stained slides, proteoglycan depletion was noted (c). High dosage of RvE1 did not reduce inflammation, cartilage erosion, bone destruction, and proteoglycan depletion (d–f) in the ankle joints of CIA mice. Original magnification of 10x and 20x.

**Figure 6:**
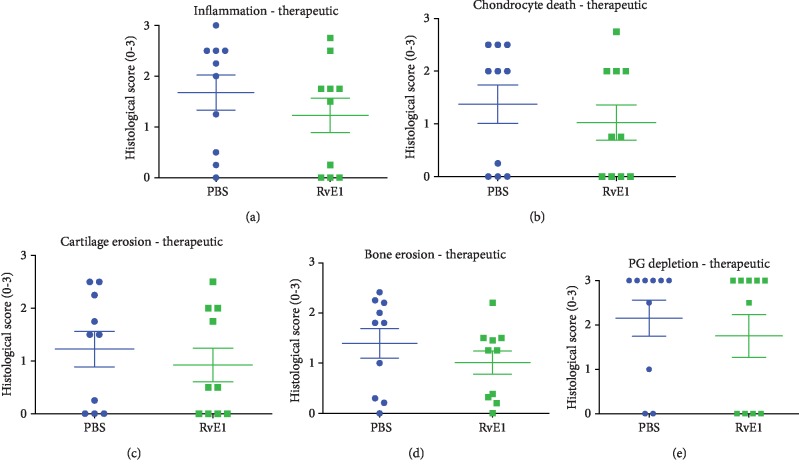
Quantification of histological findings—therapeutic. Histopathologic scores of synovial inflammation (a), chondrocyte death (b), cartilage erosion (c), bone erosion (d), and proteoglycan depletion (e) in PBS and RvE1. The parameters showed normal distribution, and therefore, Student's *t*-test was used to assess the differences between the groups. No differences were found when RvE1 was used for the treatment of CIA mice.

In the therapeutic arm of our study, histological analysis did not show differences between the PBS group (positive control) and the high dose RvE1 group (Figures 5(a)–5(f)) regarding the inflammatory process. RvE1 did not abrogate any clinical signs of disease. Quantification of histological findings showed no differences comparing PBS-treated animals with RvE1 treatment for inflammation, chondrocyte depth, cartilage erosion, bone resorption, and PG depletion (Figures 6(a)–6(e)). Taken together, our data suggest that RvE1 treatment is not an effective approach to treat CIA in DBA1/J mice in both regimens.

### 3.4. RvE1 Does Not Reduce Spontaneous Cytokine Release in Human RA Synovial Explants

**Figure 7:**
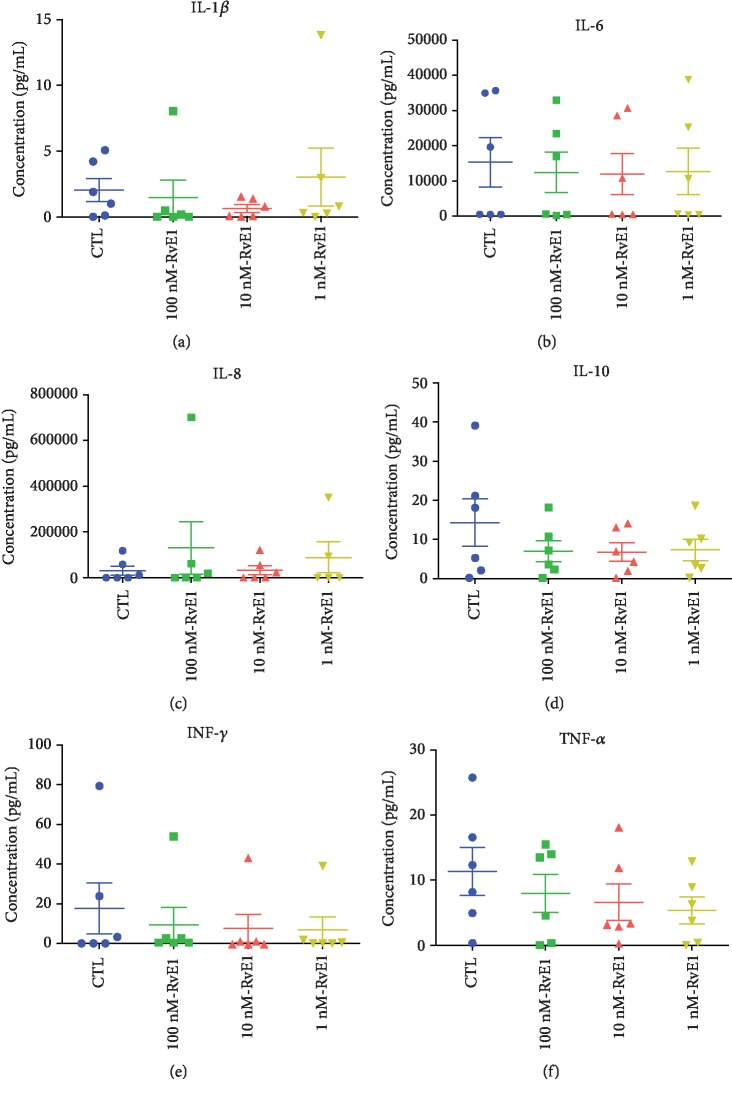
Synovial tissues from RA patients were stimulated with three different concentrations of RvE1 and with medium only (negative control) for 24 hours before supernatant collection. Six proinflammatory cytokines were measured: IL-1*β* (a), IL-6 (b), IL-8 (c), IL-10 (d), INF-*γ* (e), and TNF-*α* (f). One-way ANOVA followed by Tukey's posttest was used to access the differences among the groups. Differences were considered significant at *P* < 0.05. No differences were found among the different dosages used for all the cytokines evaluated.

To evaluate whether RvE1 suppresses spontaneous release of proinflammatory cytokines by human RA synovial explants, we incubated synovial tissue from RA patients with various concentrations of RvE1 for 24 hours. Supernatants were analyzed by Luminex for cytokine and chemokine production (Figures 7(a)–7(f), respectively). Our data demonstrated that none of the RvE1 dosages employed in this study were effective in decreasing the levels of proinflammatory cytokines compared to the control group, suggesting that RvE1 treatment is not an effective approach to treat synovial inflammation in the conditions studied.

## 4. Discussion

To date, no study has addressed the effects of systemic RvE1 treatment in experimentally induced arthritis. In RA as well as in other inflammatory conditions such as periodontitis, inflammation fails to resolve and results in chronic pathology and decreases patient quality of life. A growing body of in vitro and in vivo evidence points to the effects of RvE1 and other specialized pro-resolving mediators (SPRM) on different cell types in regulating the resolution of inflammation [12, 16, 17, 20, 33, 34]. In this context, we sought to investigate the potential beneficial effects of RvE1 aiming at decreasing disease severity in CIA mice in vivo and human synovial explant inflammation in vitro. Our findings demonstrated that systemic treatment with RvE1 in both prophylactic and therapeutic approaches did not ameliorate the disease severity at clinical and histological levels. Furthermore, utilizing different concentrations of RvE1 and synovial biopsies of RA showed no beneficial response upon RvE1 exposure, with unaffected levels of IL-1*β*, IL-6, IL-8, IL-10, INF-*γ*, and TNF-*α*.

SPRM are a family of oxylipids that include resolvins, protectins, maresins, and lipoxins [35]. SPRM arise from n-3 long-chain polyunsaturated fatty acids (n-3FA) through the action of lipoxygenase enzymes and other remodeling steps [12]. The E-series resolvins (RvE1-E3) are biosynthesized from eicosapentaenoic acid (EPA, 20 : 5 n-3) produced by hydroxyl that converts EPA to 18R-hydroxy-EPA via ASA-acetylated COX-2 as well as via second route involving p450-like reactions. 18-HEPE is then converted by 5-lipoxygenase to RvE1 and RvE2 or by 15-lipoxygenase to RvE3 [36]. RvE1 is a stereoselective agonist that interacts with at least two identified G protein-coupled receptors: chemerin receptor-23 (chemR23) and BLT1 [37]. BLT1 is expressed on neutrophils, whereas chemR23 is expressed on monocytes, macrophages, and dendritic cells and, to a lesser extent, in neutrophils and CD4+ T lymphocytes [37]. Among bone cells, bone marrow stromal cells and osteoblasts express chemR [20, 38] whereas osteoclasts express the BLT1 receptor [33]. RvE1 selectively interacts with chemR23 or BLT1 inhibiting further leukocyte infiltration and cytokine/chemokine generation, to induce the apoptosis of PMNs and their removal by macrophages and to restore tissue homeostasis.

Fish oils are a rich source of EPA that have been shown to enhance management of RA [39] and systemic lupus [40] and to reduce reoccurrence rates in Crohn's disease [41]. EPA lowers plasma triglycerides, suppresses platelet aggregation, and inhibits inflammation [42]. While a number of potential anti-inflammatory actions of n-3FA have been identified [43], it is conceivable that conversion of EPA to SPRM could be a significant contributor to the disease-mitigating effects of fish oil in inflammatory diseases. On the other hand, available data are controversial regarding the positive effects of EPA in preventing bone loss and increasing bone mineral density in in vitro and in vivo studies [33, 44, 45].

A previous study [17] demonstrated that local administration of RvE1 (4 *μ*g) significantly abrogated alveolar bone loss and diminished the number of TRAP-positive cells in a model of ligature-induced periodontal disease in rabbits showing the bone-protective action of RvE1. The same work group further revealed, using the identical animal model, that RvE1 treatment promoted resolution of inflammation and regeneration of the periodontal tissues (alveolar bone, periodontal ligament, and cement) and reduced systemic inflammatory markers C-reactive protein and IL-1*β* [16]. The recruitment and activation of neutrophils in the periodontal tissue play an important role to the bone destruction during PD [46], which seems to be comparable to neutrophil-mediated tissue damage in the RA pathogenesis. Because neutrophil degranulation contributes to the degradation of tissue in arthritis [47] and periodontal tissue (connective and bone surrounding teeth), studies investigating the histological aspects of the joints in animals with induced arthritis play a crucial role for understanding the therapeutic properties of RvE1 during the course of RA.

In view of the positive and promising effects of the RvE1 in the experimental periodontal disease model and in the atherosclerotic plaque formation both in vivo and in vitro [16, 19, 48, 49], we were intrigued to expand researches in the field of arthritis. Since RA and PD share innumerous pathological and immunological characteristics, such as the following: (1) increased infiltration of inflammatory and immune cells including neutrophils, monocytes, and T and B lymphocytes; (2) increased release of proinflammatory mediators TNF-*α*, IL-1*β*, and IL-6 and degradation enzymes (MMPs); (3) and the activation of RANK-L, we hypothesized that RvE1 treatment would have beneficial effects on arthritis progression. Surprisingly, mice treated with systemic doses of RvE1 did not decrease the severity of the disease when compared to the control group (without treatment). Only animals treated with the TNF inhibitor showed clinical improvement on the arthritis signs and symptoms. The lack of positive effects of RvE1 in our study might be explained because EPA metabolites are generated under different conditions and these metabolites, and not EPA itself, may thus be responsible for the observed biological actions [50].

A recent in vitro study [51] evaluated the effects of RvE1 on osteoclastogenesis and bone resorption to elucidate its therapeutic potential for the treatment of arthritis. RANKL-induced bone resorption was evaluated by measuring pit formation in osteoclast precursor cells (RAW264.7). The mechanisms of the inhibitory effects of RvE1 were also investigated. The authors demonstrated that RvE1 inhibited osteoclastogenesis and bone resorption, reducing the number of TRAP-positive cells, by suppressing RANKL-induced NFATc1 and c-fos expression in osteoclasts suggesting a possible therapeutic approach with RvE1 to treat rheumatoid arthritis. This finding is consistent with a previously published article by Herrera et al. [33] demonstrating that RvE1 strikingly reduced the number of differentiated osteoclasts in primary osteoclast cultures. Similarly, Gao et al. [20] showed that RvE1 modulates bone remodeling and osteoclast differentiation via chemokine-like receptor 1, rescuing osteoprotegerin production and reestablishing a satisfactory receptor activator of the NF-*κ*B ligand/OPG ratio. Due to the encouraging beneficial effects of RvE1 in decreasing proinflammatory mediators and bone markers in vitro, we sought to investigate whether RvE1 treatment would suppress spontaneous release of proinflammatory cytokines in human RA synovial explants in vitro. Our findings did not demonstrate any positive effect of different dosages of RvE1 treatment in decreasing the levels of proinflammatory cytokines. A limitation of this approach is that these types of cell cultures are limited to 24 hours due to the extensive cell death thereafter, so long-term effects of RvE1 cannot be studied in this way.

Some considerations should be mentioned when interpreting the results of the present investigation. The CIA model is a systemic and very progressive model of disease, which might have influenced the poor results achieved when RvE1 was used. Perhaps, a milder model of RA, such as the antigen-induced arthritis, with defined resolution phase would have favorable results, and local RvE1 treatment might achieve the therapeutic effects as observed in models for PD. On the other hand, even with an aggressive model such as the CIA, anti-TNF treatment but not RvE1 inhibited all signs and symptoms of the disease in the mice. Seven days after the booster injection, anti-TNF abrogates pretty much all signs of CIA in the front and rear paws, showing the effectiveness of this treatment, which corroborate previous studies in the literature [2, 27]. In view of the results achieved we would suggest (1) to use models that can discriminate more the acute versus chronic phase instead of the CIA model and (2) to apply a local instead of systemic treatment with RvE1 in a less severe arthritis model, which may shed more light into the complex molecular mechanisms involved in the resolution of synovial inflammation.

This study has some limitations that should be mentioned. Cytokine release profiles upon the application of a DAMP stimulus were not evaluated. This work is a proof of concept study aiming at elucidating if treatment with proresolving mediators possesses any biological effect in the CIA mouse model. This study will guide and shed light for future experiments in the field of arthritis and treatment with proresolving mediators and will help to develop better strategies to treat induced arthritis in mice with RvE1 based in this preliminary data. In this context, further randomized, controlled preclinical studies with different animal models of induced arthritis and different dosages/regimens of drug administration should be conducted before definitive conclusion about the CIA treatment with RvE1 could be drawn.

## 5. Conclusion

In conclusion, this study showed that systemic treatment with proresolving RvE1 was not an effective approach to treat CIA in DBA1/J mouse in both prophylactic and therapeutic strategies. Furthermore, no decrease in the expression of proinflammatory cytokines was evidenced when synovial human explants were incubated with different concentrations of RvE1. Indeed, further studies are warranted to elucidate if RvE1, alone or in combination with other regimens, is a potential candidate for RA therapy.

## Data Availability

The data used to support the findings of this study are available from the corresponding author upon request.
